# Exploration of the relationship between tumor mutation burden and immune infiltrates in colon adenocarcinoma

**DOI:** 10.7150/ijms.51918

**Published:** 2021-01-01

**Authors:** Rong Ouyang, Zhongzhuan Li, Peng Peng, Jinxiu Zhang, Jun Liu, Mengbin Qin, Jiean Huang

**Affiliations:** 1 Department of Gastroenterology, The Second Affiliated Hospital of Guangxi Medical University, Nanning, China; 2 Department of Gastroenterology, Liuzhou Worker's Hospital, Liuzhou, China

**Keywords:** Tumor mutation burden (TMB), colon adenocarcinoma (COAD), immune signature, prognosis

## Abstract

**Background:** Tumor mutation burden (TMB) was correlated with the immunotherapeutic response in various malignancies. We aimed to evaluate the TMB immune signature in colon adenocarcinoma (COAD).

**Methods:** Gene expression profile, mutation and clinical data of COAD patients were obtained from The Cancer Genome Atlas (TCGA) database. The samples were divided into high and low TMB level groups to identify differentially expressed genes (DEGs). Functional enrichments analyzes were performed to identify the biological functions of the DEGs. Then, immune cell infiltration signatures were calculated by the CIBERSORT algorithm. Finally, Cox proportional hazard model was constructed to estimate the prognostic value of the identified immune-related genes.

**Results:** Gene set enrichment analysis in the high-TMB level group showed that DEGS were enriched in immune-related pathways, such as antigen processing and presentation, Toll-like receptor signaling and natural killer cell-mediated cytotoxicity. A higher infiltration level of CD8+ T cells, CD4+ T cells, activated NK cells , M1 Macrophages and T follicular helper cells was observed in the high-TMB level group. Furthermore, a Cox regression model combined with survival analysis based on the expression level of four identified prognostic genes was constructed, validated anf revealed that higher risk-score levels conferred poor survival outcomes in COAD patients.

**Conclusions:** Our data demonstrate that the high TMB levels are associated with an immune signature in COAD and deepen the molecular understanding of TMB function in tumor immunotherapy.

## Introduction

Colorectal cancer (CRC) is one of the most common malignances in the digestive system and a major cause of cancer-related mortality worldwide 1. The 2018 Colorectal Cancer Statistics estimated that almost 1.1 million new cases of colorectal cancer are diagnosed and 551,269 deaths occur each year worldwide 2. Although the 5-year survival rate reaches aproximately 90% in the early stage patients, in the metastatic stage this rate is reduced to 13% 3. Therefore, it is urgent to find more efficient therapies than current ones for patients with invasive tumors.

Colon adenocarcinoma (COAD) is the leading type of CRC and is developed from adenomatous lesions that evolve into invasive carcinomas due to the accumulated genetic mutations 4. Mutations in mismatch repair genes result in failure to recognize and correct replication errors in the DNA synthesis process, eventually affecting the genes involved in apoptosis or in the cell cycle regulation and thus leading to neoplastic changes 5. However, recent. studies have shown that mutations transcribed and translated into tumor cells can form new antigens that can be recognized and targeted by the immune system^6^. Moreover, immunotherapy has shown proven effectiveness in the treatment of advanced and aggressive tumors 7, 8, such as programmed death-1 (PD-1) inhibitors, which has been shown to present a remarkable and durable response in CRC 7, 9.

Recently, an emerging biomarker called as tumor mutation burden (TMB) had been discovered. TMB has proved to be a realible biomarker for immunotherapy treatments in many tumor types. Patients with higher TMB levels tend to become more immunogenic and have an improved response to immune checkpoint blockades 7, 10. However, the molecular mechanism of TMB in immunotherapy is still unknown and needs to be explored. The Cancer Genome Atlas (TCGA) database provides several expression profiles and mutation data for different types of cancer, as well as corresponding clinical data. The present study aimed to evaluate the potential immune signature of TMB in COAD.

## Materials and methods

### Database and genomic analysis

Somatic mutation data (mutect. Somatic. Maf), gene expression files and clinical data of COAD patients were downloaded from the TCGA database (https://tcga-data.nci.nih.gov/tcga/). The mutation data was analyzed and visualized using “maftools” package from R 3.6.0 11. TMB was defined as the total amount of somatic variants per megabase (MB) of genome. According to the TMB level, COAD samples were classified into low-TMB level (<20 mutations per MB) and high-TMB level groups (≥20 mutations per MB) 10, 12-14.

### Differentially expressed genes and functional analysis

The low- and high-TMB groups were divided using the R software (3.6.0). The “limma” package was used to screen the differentially expressed genes (DEGs) in the two groups using False Discovery Rate (FDR) <0.05 and Fold Change (FC) > 1.5. Heatmaps were visualized using “pheatmap” package and “ClusterProfiler” package was used for functional analysis. Gene Ontology (GO) and the Kyoto Encyclopedia of Genes and Genomes (KEGG) pathway terms were considered statistically significant when FDR<0.01. Besides, the gene set enrichment analysis (GSEA) was performed using the GSEA software, which obtained data sets from the MSigDB database (http://software.broadinstitute.org/gsea/msigdb/), and FDR < 0.05 as cutoff. In addition, a total of 2,498 immune-related genes (IRGs; Supplementary Table S1) were downloaded from the Immunology Database and Analysis Portal (ImmPort) database (https://www. immport.org/home) 15 and the differentially expressed immune genes (DEIGs) were obtained using the “VennDiagram” package.

### Correlation analysis of immune infiltration analysis

The CIBERSORT algorithm^16^ was used to calculate the proportion of infiltrating immune cells in COAD samples using LM22 as signature matrix file. To enhance the robustess of the results, CIBERSORT uses Monte Carlo sampling based on microarray data to derive the deconvoluted P value for each samples. At a threshold of P <0.05, the results of the proportion of the infiltrating immune cells were considered with high accuracy. Wilcoxon rank-sum test was performed to analyze the differential abundances of infiltrating immune cells between low- and high-TMB level groups, which were visualized using the “vioplot” package.

### Cox proportional hazards model construction and model validation

Univariate Cox proportional hazard regression analysis of differentially expressed immune genes (DEIGs) was employed to discover the prognostic genes by using the “survival” R package and Coxph function. The adjusted P-value < 0.05 was considered as the significance cutoff. Then, the expression level of the identified prognostic genes was combined with their regression coefficients (β) and fitted in a multivariate Cox regression analysis, resulting in a risk model that presentes the following formula: Risk score = expression_gene1_ × β_gene 1_ + expression_gene 2_ × β_gene 2_ + ... expression_gene n_ ×β_gene n_. Patients were divided into low-risk and high-risk groups based on the median value of risk score. Kaplan-Meier analysis was performed to evaluate the survival status of these two groups. Receiver operating characteristic (ROC) curve were conducted by using the “survivalROC” R package. In addition, patients were also divided into low- and high-gene expression groups, according to the average of prognostic gene expression data, and the survival rate between these groups was compared using Kaplan-Meier analysis.

### Statistical analysis

All the statistical data analyses were performed using the R software (version 3.6.0). P < 0.05 was considered statistically significant.

## Results

### Landscape of mutation profiles in COAD

**Figure 1:**
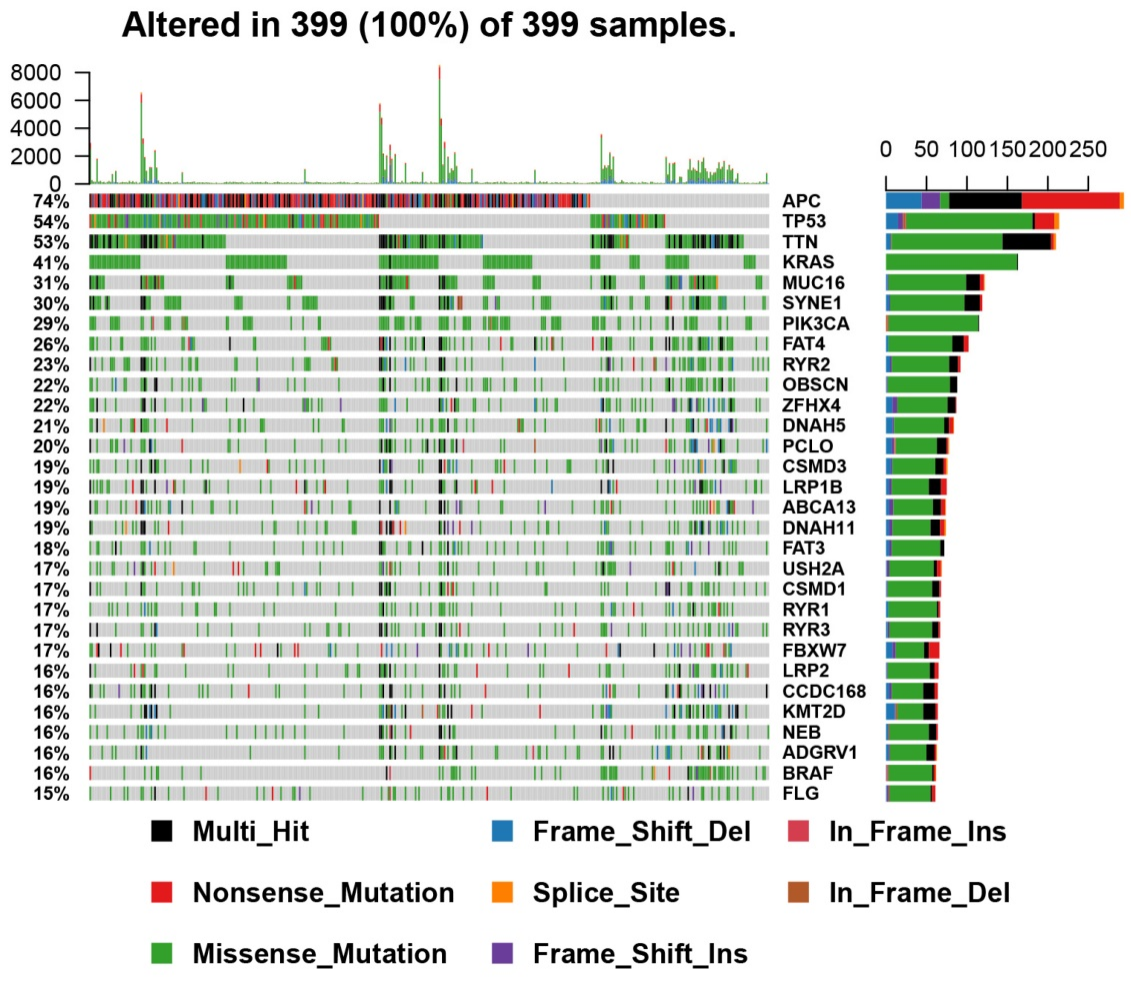
Landscape of mutation profiles in COAD samples. Mutation information of each gene in each sample was shown in the waterfall plot, in which various colors with annotations at the bottom represented the different mutation types. The barplot above the legend exhibited the mutation burden.

**Figure 2:**
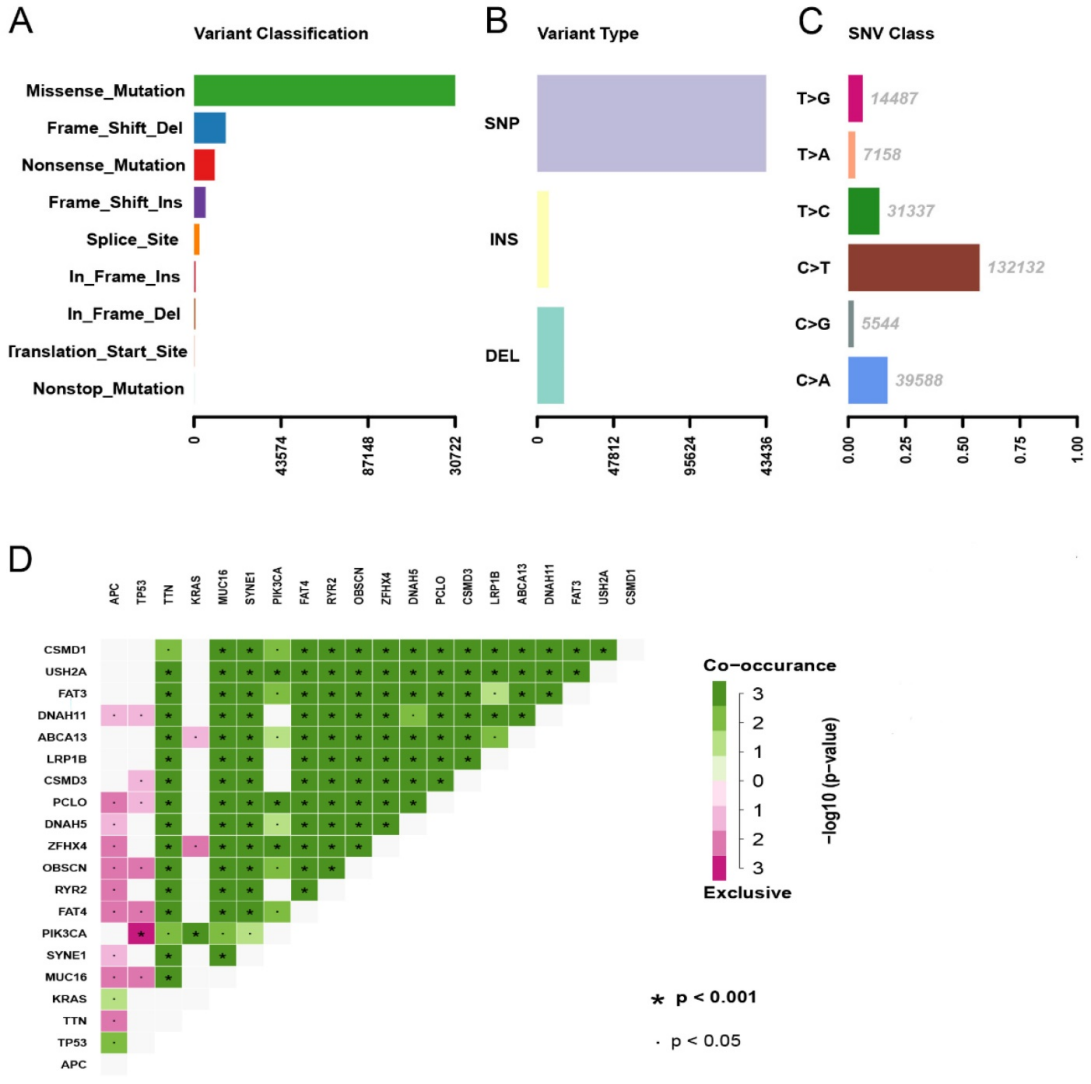
Summary of the mutation information with statistical calculations. (A, B) Variant classification and type of genetic alterations in COAD. (C) The SNV class of COAD; (D) The coincident and exclusive associations across mutated genes. SNP, single nucleotide polymorphism; SNV, single nucleotide variants.

A total of 399 COAD samples with somatic mutation profiles were downloaded from the TCGA database. Waterfall plot shows the gene mutation information in all obtained samples (Figure 1). TTN, APC, MUC16, SYNE1, TP53, FAT4, RYR2, KRAS, OBSCN, PIK3CA proved to be the top 10 mutated genes. According to the different mutation classification categories, the missense mutation was the one that obtained the highest proportion (Figure 2A) and C>T substitution single nucleotide polymorphism (SNP) was the one that occurred most frequently (Figures 2B and C) in the COAD samples. In addition, a correlation matrix of mutated genes is presented, in which green represents the co-occurrence relationship, while red represents mutual exclusion (Figure 2D).

### Gene expression profiles comparation and functional enrichment in different TMB groups

**Figure 3:**
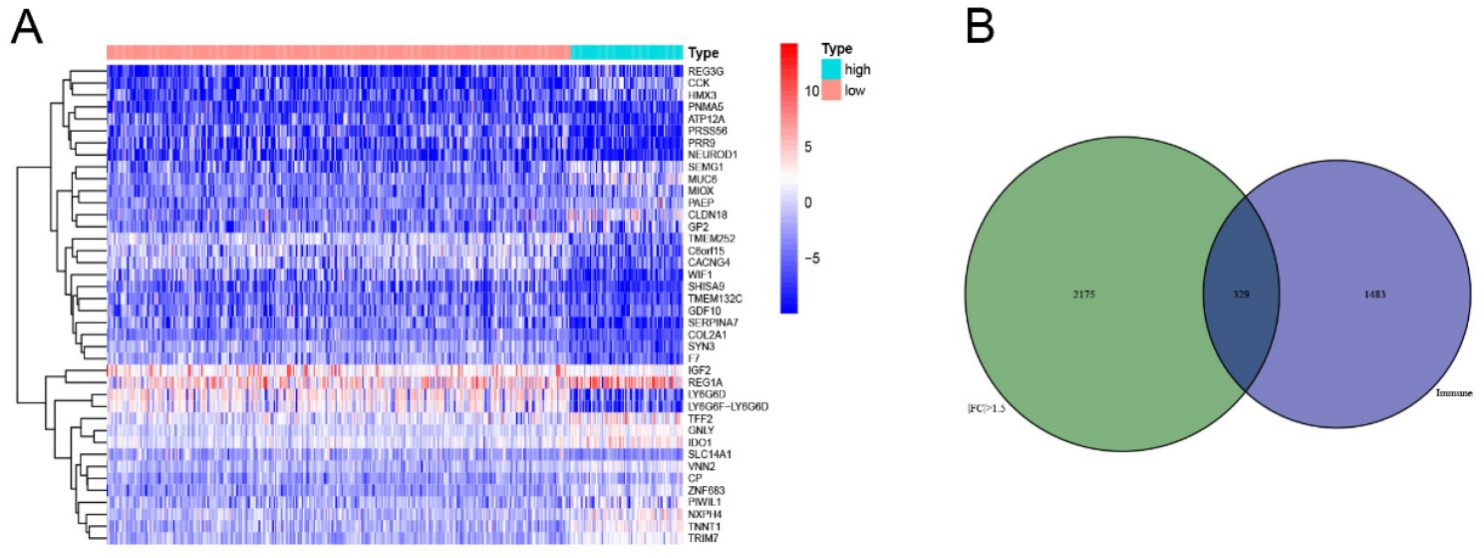
Comparisons of gene expression profiles in low- and high-TMB samples. (A) Top 30 DEGs were shown in heatmap plot; (B) Identification of TMB-related immune genes.

**Figure 4:**
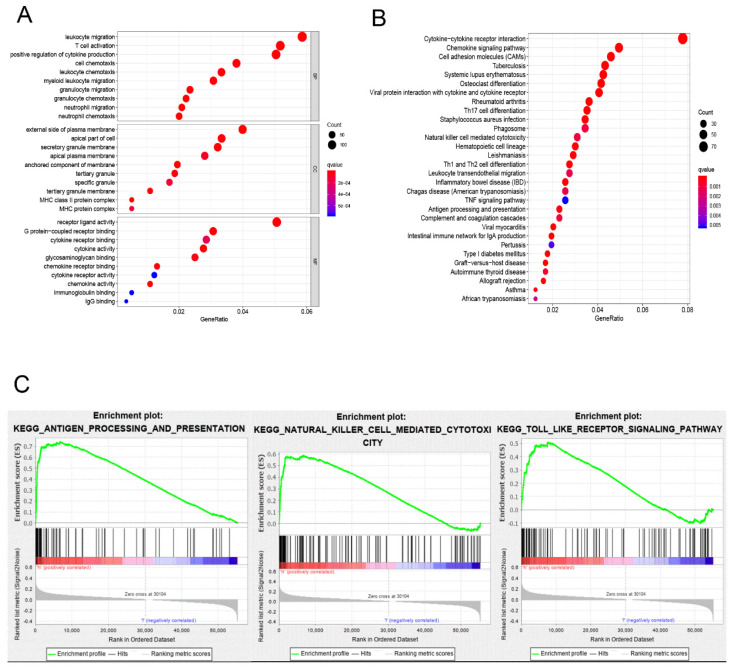
Functional enrichment in different TMB groups. (A,B) Functional enrichment pathway analysis of TMB-related DEGs in COAD, including biological processes (BP), cellular components (CC), molecular function (MF), and KEGG pathway. (C) Results of significantly enriched pathways in the high-TMB group by GESA. Enrichment scores (ES, green line) indicate the degree to which the genome is overexpressed at the top or bottom of the list of sequenced genes.

The division of the COAD samples into two groups, according to the TMB level, resulted in 323 samples classified as low-TMB level cases and 76 as high-TMB level cases. A total of 2504 DEGs were identified between the low- and high-TMB level groups based on the screening criteria (FDR <0.05 and FC >1.5). The top 30 significant DEGs were visualized on a heatmap (Figure 3A). Then, GO and KEGG enrichment analyzes were conducted to explore the biological roles of the identified DEGs. GO enrichment analysis showed that the DEGs were mainly enriched in the biological process (BP) terms associated with T cell activation, positive regulation of cytokine production, migration and chemotaxis of inflammatory cells including leukocytes, granulocytes and neutrophils (Figure 4). KEGG enrichment pathway analysis revealed that DEGs mainly participated in following pathways: cytokine-cytokine receptor interaction, chemokine signaling, cell adhesion molecules (CAMs), Th17, T1 and T2 cell differentiation, natural killer cell-mediated cytotoxiccity and immune-related diseases (Figures 4A and,B). GSEA analysis in the high-TMB level group showed that genes were enriched in several pathways, including immune-related pathways, such as antigen processing and presentation, Toll-like receptor signaling and natural killer cell-mediated cytotoxicity (Figure 4C). All significantly enriched pathways (P<0.05) are listed in Supplementary Table 1.

Therefore, GO, KEGG ands GSEA enrichment analyzes indicated that TMB level is correlated with an immune signature in the COAD. Then,329 overlapped genes were identified between DEGs in the different TMB level groups and the IRGs obtained from ImmPort database. The overlapped genes were represented in Venn diagram (Figure 3B) and listed in Supplementary Table 2.

### Correlation of TMB with immune signatures and prognosis in COAD patients

**Figure 5:**
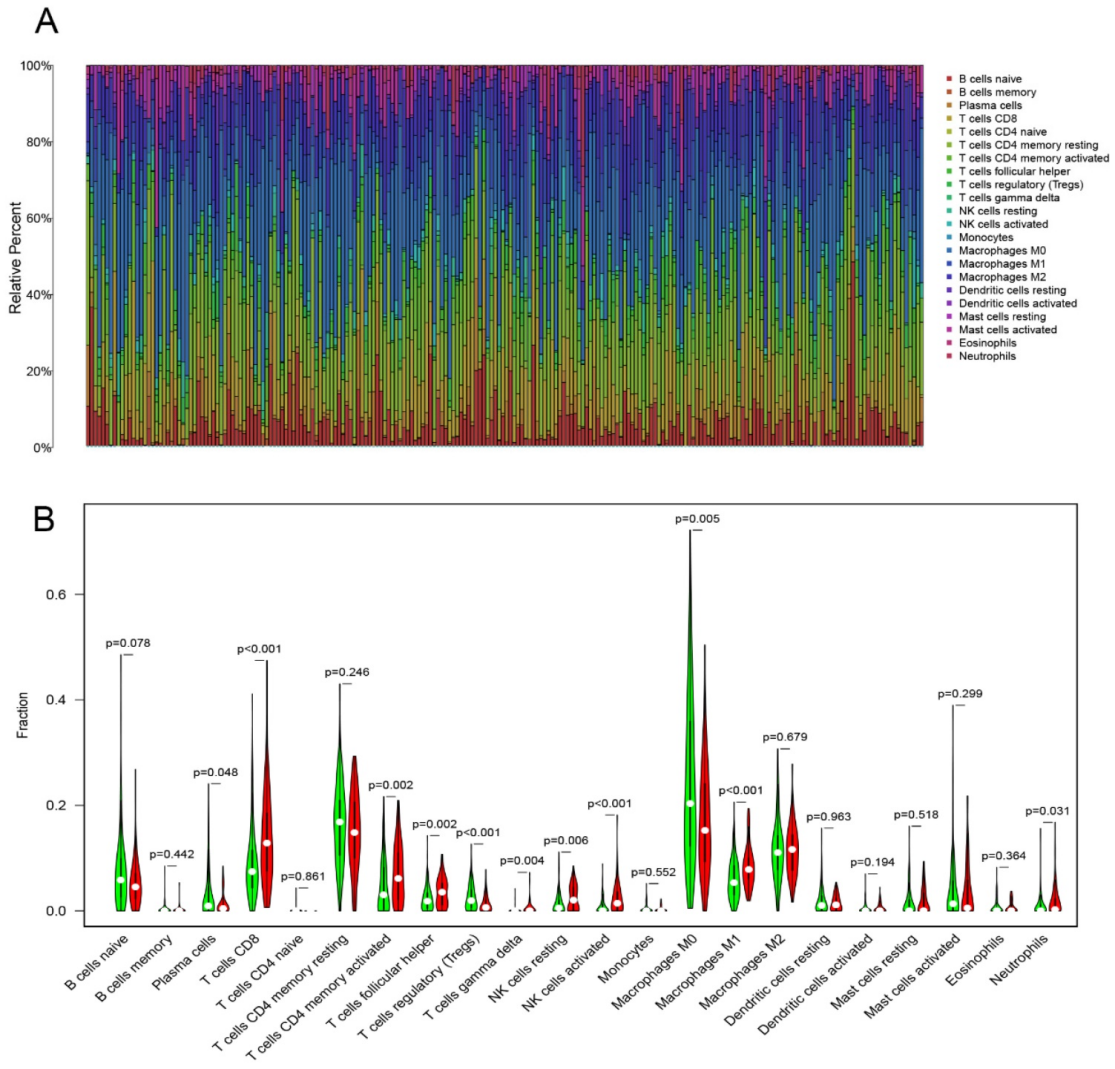
Immune cell infiltrations in COAD. (A) The specific 22 immune fractions represented by various colors in each sample were shown in barplot. (B) Comparisons of 22 immune cell infiltrations between the high‐TMB and low‐TMB groups.

CIBERSORT algorithm calculated the proportion of 22 infiltrating immune cells (threshold of P < 0.05) in the COAD samples (Figure 5A). Then, Wilcoxon rank-sum test was used to reveal the different proportions of infiltrating immune cells between the low- and high-TMB level samples. High-TMB level group presented a significant higher infiltration levels of CD8+ T cells, CD4+ T cells memory, activated NK cells , M1 macrophages, resting NK cells, T follicular helper cells and gamma delta T cells than the low-TMB group. On the other hand, the low-TMB level group showed a higher infiltration level of regulatory T cells (Tregs), plasma cells and M0 macrophages (Figure 5B). Next, we analyzed the relationship between TMB and survival outcomes, and Kaplan-Meier survival analysis revealed that the high- and low-TMB groups had no statistically difference with the survival rate (Supplementary Figure 1).

### Construction of the Cox regression model and survival analysis

**Figure 6:**
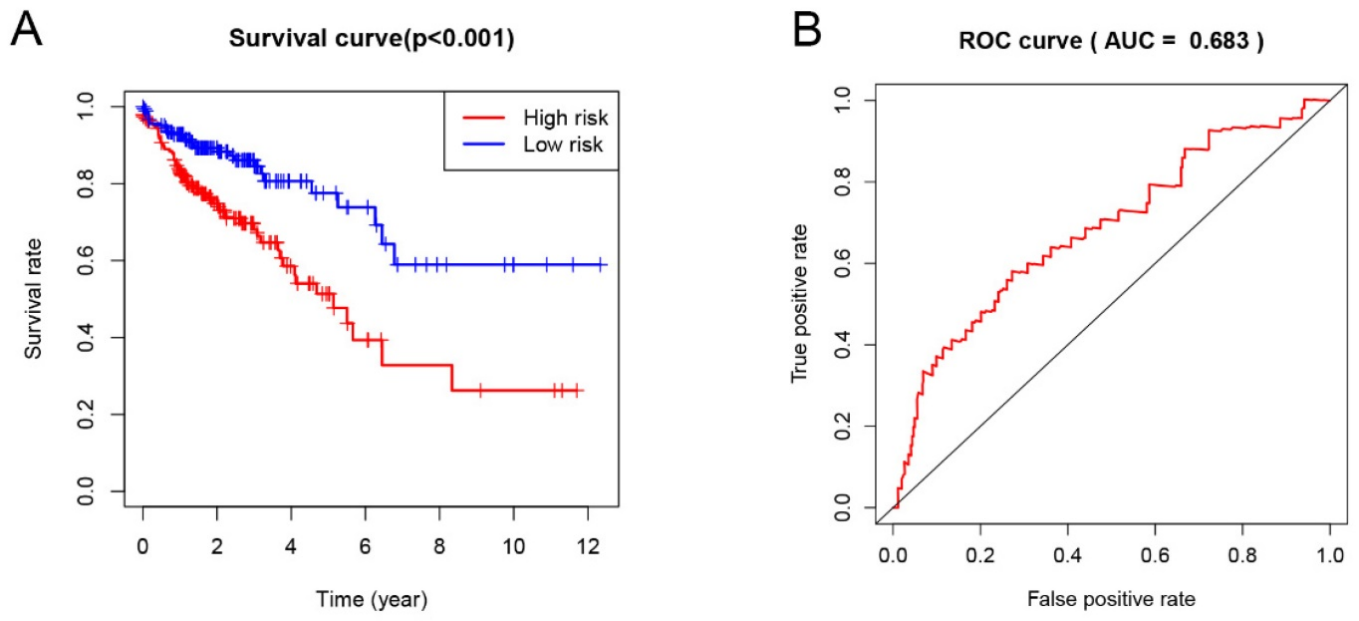
Prognostic analysis based on risk score model of the 4 genes. (A) Kaplan-Meier curves for the low- and high-risk groups; (B) The receiver operating characteristic (ROC) curve validation of prognostic value by the risk score.

**Figure 7:**
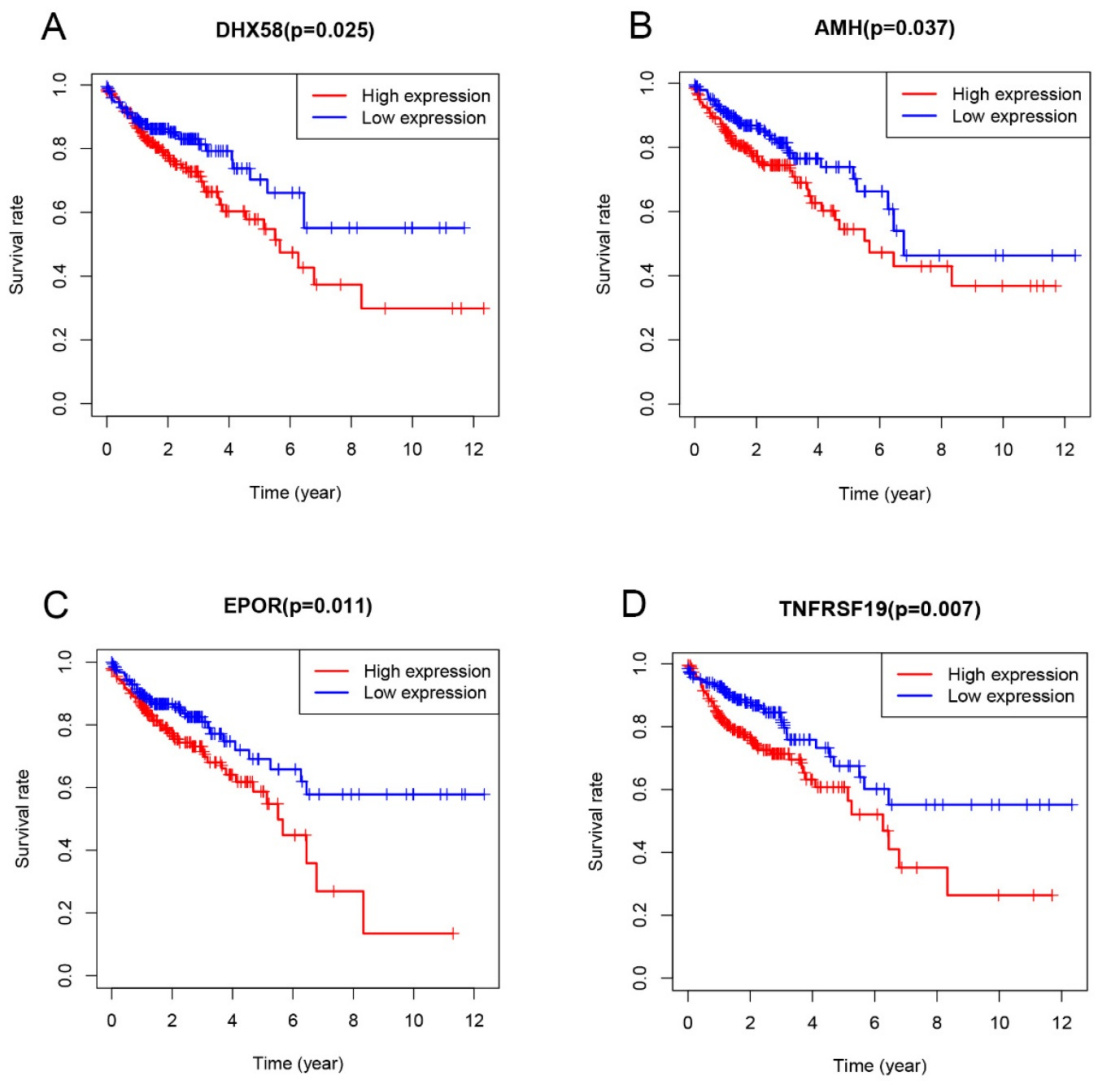
Evaluation of the prognostic value of the four-gene signature using Kaplan-Meier analysis.

Univariate Cox proportional hazard regression analysis was performed to discover the prognostic genes based on 329 overlapping genes between DEGs and IRGs. The results indicated DHX58, AMH, EPOR and TNFRSF19 as COAD prognostic genes. Then, multivariable Cox proportional hazards regression was utilized to construct a prognostic model as follows: risk score = (0.083 × exp DHX58) + (0.058 × exp AMH) + (0.156 × exp EPOR) + (0.082 × exp TNFRSF19). Thus, COAD patients were divided into low- and high-risk groups, according to the median cutoff value of the risk score. The survival risk curve indicates that patients in the high-risk group have a significantly shorter overall survival time than patients in the low-risk group (P<0.001; Figure 6A). The ROC curve presented a reliable ability of the survival prediction based on the prognostic model and the AUC was 0.683 (Figure 6B). Survival analysis of prognostic genes reveal that high expression levels of DHX58, AMH, EPOR and TNFRSF19 were correlated with poor survival outcomes in COAD patients (P<0.05; Figure 7).

## Discussion

CRC is a highly heterogeneous cancer type at the genetic and molecular levels, and therefore susceptible to respond to immunotherapy. Immune checkpoint inhibitors (ICIs) have been found to be effective for the treatment of some subsets of CRC presenting heavily mutated tumors that are mismatch repair-deficient (MMR-D) and microsatellite instability-high (MSI-H) 17. TMB is characterized as enriched with neoantigens and contributes to activate the antitumor immune response 18. A high TMB level is considered to be closely associated with MMR-D and MSI-H. An analysis of the tumor landscape reveals that more than 80% of samples with a high MSI level display a high level of TMB (>20 mutations/Mb) 19. In addition, another study has shown that 90% of MMR-D tumors also exhibit a high TMB level 14.

In this study, we showed the transcriptome profile comparison between COAD patients classified according to the different TMB levels (low and high) and who presented distinct biological features. GO analysis indicated that the DEGs between these groups were mainly enriched in immune inflammatory response processes. In addition, KEGG enrichment pathway analysis found DEGs participate in chemokine signaling and cell adhesion molecules pathways, which are associated with cancer immunology. Chemokines participate in the tumor occurrence and metastasis, regulating cell proliferation, activation, migration and differentiation through the chemokine signaling pathway 20, 21. Furthermore, GSEA analysis in the high-TMB level group showed that identified DEGs were enriched in different immune-related pathways, including antigen processing and presentation, Toll-like receptor signaling and natural killer cell-mediated cytotoxicity. Previous studies have suggested that antigen presentation is an essential procedure for presenting the tumor antigen of malignant cells to T cells 22. NK cells, in turn, play a role in cancer immuno-oncology because of their inherent qualities that they can introduce antigen specificity by genetic modification 23, 24. Finally, the Toll-like receptor signaling pathway showed an inflammatory response that activates innate and adaptive immunity, which plays a fundamental role in epithelial proliferation, apoptotic response and barrier homeostasis of CRC 25, 26.

Immune-cell infiltration is considered one of the carcinoma typical features and it can reflect the growth and migration of tumor cells and the effect of treatment on them 27. High-TMB level group displayed a significant higher infiltration levels of CD8+ T cells, CD4+ T cells, NK cells, M1 macrophages and T follicular helper cells. Remarkably, these immune cells are found to be at a high level in MSI-rich CRC samples 28, 29. In addition, previous studies have reported that there was a higher density of CD8 + T cells in the PD-1 blockade responsive samples of various tumor types 30. The manipulation of tumor infiltrating NK cells plays a critical role in initiating a multilayered antitumor response 31. A previous study conducted on mouse model showed that the depletion of NK cells eliminated the PD-1 ligand inhibition functions 32. Then, M1 Macrophages can promote CD8 + T cell activation through cytokine secretion and antigen presentation, which is essential for immunotherapy efficacy 33. In addition, this macrophage infiltration in tumors may increase the survival of patients treated with PD-1 blockade therapies 34.

Multivariate Cox regression analysis established a prognostic risk scoring model for COAD based on the expression of 4 prognostic immune genes identified here, namely AMH, DHX58, EPOR and TNFRSF19. These genes presented a negative correlation with survival in COAD. DHX58, also known as LGP2 (laboratory of genetics and physiology 2), is a negative regulator in the RIG-1 signalling pathway 35, 36. Although there are still few studies on the DHX58 role in tumors, RIG-1 signaling pathway activation, which results in the stimulation of cytotoxic immune cells 37, 38, showed prospects for development in cancer immunotherapy when combined with immune checkpoint inhibitors in clinical trials. AMH, a member of the transforming growth factor (TGF-β) family 39, has been found associated with the regulation of epithelial-mesenchymal transition (EMT) 40. AMH mutations has been identified in esophageal squamous cell carcinoma and gastric cancer 41. EPOR can act far beyond erythropoiesis and have an immunoregulatory effect on several immune cells including T and B lymphocytes, macrophages, mast cells and dendritic cells 42. Recently, it has also been shown to be essential for tumor proliferation and survival 43, 44. TNFRSF19 belongs to the tumor necrosis factor (TNF) receptor superfamily and it has been identified that the up-regulation of TNFRSF19 is associated with poor outcomes in various types of cancer 45-47. In addition, it has been shown that TNFRSF19 may be involved in the dysregulation of β-catenin activity that can result in the development of colorectal cancers 48.

Finally, the prognostic model constructed based on the prognostic immune genes showed a reliable ability to predict survival. Patients who had high risk-score values presented a worse survival time than the those who had low values. As far as we known, this is the first study that evaluates the correlation between TMB and CRC that uses 20 mutations per MB as TMB cutoff value.

## Conclusion

To summarize, this study provides a systematic and comprehensive analysis to stratify COAD samples into different TMB level groups with different biological phenotypes. The correlation between TMB and immune cell infiltration signatures in CRC was evaluated and it was obtained a list of TMB related genes that can influence the prognosis in colon cancer. We believe that the results obtained in this work can have implications for the development of new immunological therapeutic strategies for CRC.

## Supplementary Material

Supplementary figure and tables.

## Author Contributions

The study was designed by R.O., J.H. and Z.L. designed this study, R.O. and J.Z. extracted the data. R.O. and P.P. analyzed the data, R.O., M.Q. and J.L. wrote and revised the article; all authors commented on drafts of the manuscript. All authors have approved the final draft of the manuscript.

## Data Availability

The data used to support the findings of this study are available from the corresponding author upon request.

## References

[1] Siegel RL, Miller KD, Goding Sauer A, Fedewa SA, Butterly LF, Anderson JC (2020). Colorectal cancer statistics, 2020. CA: a cancer journal for clinicians.

[2] Araghi M, Soerjomataram I, Jenkins M, Brierley J, Morris E, Bray F (2019). Global trends in colorectal cancer mortality: projections to the year 2035. International journal of cancer.

[3] Mattiuzzi C, Sanchis-Gomar F, Lippi G (2019). Concise update on colorectal cancer epidemiology. Annals of translational medicine.

[4] Mutch MG (2007). Molecular profiling and risk stratification of adenocarcinoma of the colon. Journal of surgical oncology.

[5] Haraldsdottir S, Hampel H, Tomsic J, Frankel WL, Pearlman R, de la Chapelle A (2014). Colon and endometrial cancers with mismatch repair deficiency can arise from somatic, rather than germline, mutations. Gastroenterology.

[6] Riaz N, Morris L, Havel JJ, Makarov V, Desrichard A, Chan TA (2016). The role of neoantigens in response to immune checkpoint blockade. International immunology.

[7] Le DT, Uram JN, Wang H, Bartlett BR, Kemberling H, Eyring AD (2015). PD-1 Blockade in Tumors with Mismatch-Repair Deficiency. The New England journal of medicine.

[8] Omar HA, Tolba MF (2019). Tackling molecular targets beyond PD-1/PD-L1: Novel approaches to boost patients' response to cancer immunotherapy. Critical reviews in oncology/hematology.

[9] Llosa NJ, Cruise M, Tam A, Wicks EC, Hechenbleikner EM, Taube JM (2015). The vigorous immune microenvironment of microsatellite instable colon cancer is balanced by multiple counter-inhibitory checkpoints. Cancer discovery.

[10] Rizvi NA, Hellmann MD, Snyder A, Kvistborg P, Makarov V, Havel JJ (2015). Cancer immunology. Mutational landscape determines sensitivity to PD-1 blockade in non-small cell lung cancer. Science (New York, NY).

[11] Mayakonda A, Lin DC, Assenov Y, Plass C, Koeffler HP (2018). Maftools: efficient and comprehensive analysis of somatic variants in cancer. Genome research.

[12] Hatakeyama K, Nagashima T, Urakami K, Ohshima K, Serizawa M, Ohnami S (2018). Tumor mutational burden analysis of 2,000 Japanese cancer genomes using whole exome and targeted gene panel sequencing. Biomedical research (Tokyo, Japan).

[13] Liang WS, Vergilio JA, Salhia B, Huang HJ, Oki Y, Garrido-Laguna I (2019). Comprehensive Genomic Profiling of Hodgkin Lymphoma Reveals Recurrently Mutated Genes and Increased Mutation Burden. The oncologist.

[14] Stadler ZK, Battaglin F, Middha S, Hechtman JF, Tran C, Cercek A (2016). Reliable Detection of Mismatch Repair Deficiency in Colorectal Cancers Using Mutational Load in Next-Generation Sequencing Panels. Journal of clinical oncology: official journal of the American Society of Clinical Oncology.

[15] Bhattacharya S, Andorf S, Gomes L, Dunn P, Schaefer H, Pontius J (2014). ImmPort: disseminating data to the public for the future of immunology. Immunologic research.

[16] Newman AM, Liu CL, Green MR, Gentles AJ, Feng W, Xu Y (2015). Robust enumeration of cell subsets from tissue expression profiles. Nature methods.

[17] Ganesh K, Stadler ZK, Cercek A, Mendelsohn RB, Shia J, Segal NH (2019). Immunotherapy in colorectal cancer: rationale, challenges and potential. Nature reviews Gastroenterology & hepatology.

[18] Topalian SL, Taube JM, Anders RA, Pardoll DM (2016). Mechanism-driven biomarkers to guide immune checkpoint blockade in cancer therapy. Nature reviews Cancer.

[19] Chalmers ZR, Connelly CF, Fabrizio D, Gay L, Ali SM, Ennis R (2017). Analysis of 100,000 human cancer genomes reveals the landscape of tumor mutational burden. Genome medicine.

[20] Mukaida N, Baba T (2012). Chemokines in tumor development and progression. Experimental cell research.

[21] Itatani Y, Kawada K, Inamoto S, Yamamoto T, Ogawa R, Taketo MM (2016). The Role of Chemokines in Promoting Colorectal Cancer Invasion/Metastasis. International journal of molecular sciences.

[22] Sánchez-Paulete AR, Teijeira A, Cueto FJ, Garasa S, Pérez-Gracia JL, Sánchez-Arráez A (2017). Antigen cross-presentation and T-cell cross-priming in cancer immunology and immunotherapy. Annals of oncology: official journal of the European Society for Medical Oncology.

[23] Davies JOJ, Stringaris K, Barrett AJ, Rezvani K (2014). Opportunities and limitations of natural killer cells as adoptive therapy for malignant disease. Cytotherapy.

[24] Rezvani K, Rouce R, Liu E, Shpall E (2017). Engineering Natural Killer Cells for Cancer Immunotherapy. Molecular therapy: the journal of the American Society of Gene Therapy.

[25] Sato Y, Goto Y, Narita N, Hoon DS (2009). Cancer Cells Expressing Toll-like Receptors and the Tumor Microenvironment. Cancer microenvironment: official journal of the International Cancer Microenvironment Society.

[26] Bortoluci KR, Medzhitov R (2010). Control of infection by pyroptosis and autophagy: role of TLR and NLR. Cellular and molecular life sciences: CMLS.

[27] Nagarsheth N, Wicha MS, Zou W (2017). Chemokines in the cancer microenvironment and their relevance in cancer immunotherapy. Nature reviews Immunology.

[28] Becht E, de Reyniès A, Giraldo NA, Pilati C, Buttard B, Lacroix L (2016). Immune and Stromal Classification of Colorectal Cancer Is Associated with Molecular Subtypes and Relevant for Precision Immunotherapy. Clinical cancer research: an official journal of the American Association for Cancer Research.

[29] Guinney J, Dienstmann R, Wang X, de Reyniès A, Schlicker A, Soneson C (2015). The consensus molecular subtypes of colorectal cancer. Nature medicine.

[30] Herbst RS, Soria JC, Kowanetz M, Fine GD, Hamid O, Gordon MS (2014). Predictive correlates of response to the anti-PD-L1 antibody MPDL3280A in cancer patients. Nature.

[31] Chiossone L, Dumas PY, Vienne M, Vivier E (2018). Natural killer cells and other innate lymphoid cells in cancer. Nature reviews Immunology.

[32] Hsu J, Hodgins JJ, Marathe M, Nicolai CJ, Bourgeois-Daigneault MC, Trevino TN (2018). Contribution of NK cells to immunotherapy mediated by PD-1/PD-L1 blockade. The Journal of clinical investigation.

[33] Murray PJ, Wynn TA (2011). Protective and pathogenic functions of macrophage subsets. Nature reviews Immunology.

[34] Liu Y, Zugazagoitia J, Ahmed FS, Henick BS, Gettinger SN, Herbst RS (2020). Immune Cell PD-L1 Colocalizes with Macrophages and Is Associated with Outcome in PD-1 Pathway Blockade Therapy. Clinical cancer research: an official journal of the American Association for Cancer Research.

[35] Saito T, Hirai R, Loo YM, Owen D, Johnson CL, Sinha SC (2007). Regulation of innate antiviral defenses through a shared repressor domain in RIG-I and LGP2. Proceedings of the National Academy of Sciences of the United States of America.

[36] Vitour D, Meurs EF (2007). Regulation of interferon production by RIG-I and LGP2: a lesson in self-control. Science's STKE: signal transduction knowledge environment.

[37] Poeck H, Besch R, Maihoefer C, Renn M, Tormo D, Morskaya SS (2008). 5'-Triphosphate-siRNA: turning gene silencing and Rig-I activation against melanoma. Nature medicine.

[38] Besch R, Poeck H, Hohenauer T, Senft D, Häcker G, Berking C (2009). Proapoptotic signaling induced by RIG-I and MDA-5 results in type I interferon-independent apoptosis in human melanoma cells. The Journal of clinical investigation.

[39] Massagué J (2012). TGFβ signalling in context. Nature reviews Molecular cell biology.

[40] Zheng X, Carstens JL, Kim J, Scheible M, Kaye J, Sugimoto H (2015). Epithelial-to-mesenchymal transition is dispensable for metastasis but induces chemoresistance in pancreatic cancer. Nature.

[41] Hu N, Kadota M, Liu H, Abnet CC, Su H, Wu H (2016). Genomic Landscape of Somatic Alterations in Esophageal Squamous Cell Carcinoma and Gastric Cancer. Cancer research.

[42] Lisowska KA, Debska-Slizień A, Bryl E, Rutkowski B, Witkowski JM (2010). Erythropoietin receptor is expressed on human peripheral blood T and B lymphocytes and monocytes and is modulated by recombinant human erythropoietin treatment. Artificial organs.

[43] Wu P, Zhang N, Wang X, Zhang C, Li T, Ning X (2012). The erythropoietin/erythropoietin receptor signaling pathway promotes growth and invasion abilities in human renal carcinoma cells. PloS one.

[44] Ke S, Chen S, Dong Z, Hong CS, Zhang Q, Tang L (2017). Erythrocytosis in hepatocellular carcinoma portends poor prognosis by respiratory dysfunction secondary to mitochondrial DNA mutations. Hepatology (Baltimore, Md).

[45] Spanjaard RA, Whren KM, Graves C, Bhawan J (2007). Tumor necrosis factor receptor superfamily member TROY is a novel melanoma biomarker and potential therapeutic target. International journal of cancer.

[46] Paulino VM, Yang Z, Kloss J, Ennis MJ, Armstrong BA, Loftus JC (2010). TROY (TNFRSF19) is overexpressed in advanced glial tumors and promotes glioblastoma cell invasion via Pyk2-Rac1 signaling. Molecular cancer research: MCR.

[47] Fafilek B, Krausova M, Vojtechova M, Pospichalova V, Tumova L, Sloncova E (2013). Troy, a tumor necrosis factor receptor family member, interacts with lgr5 to inhibit wnt signaling in intestinal stem cells. Gastroenterology.

[48] Schön S, Flierman I, Ofner A, Stahringer A, Holdt LM, Kolligs FT (2014). β-catenin regulates NF-κB activity via TNFRSF19 in colorectal cancer cells. International journal of cancer.

