# Supplementary material for: Exploration of the relationship between tumor mutation burden and immune infiltrates in colon adenocarcinoma
Source: Int J Med Sci. 2021 Jan 1;18(3):685–94. doi: 10.7150/ijms.51918 (PMC7797534; doi:10.7150/ijms.51918)

Supplementary Table 1. GSEA Pathway Enrichment Analysis in the high-TMB level group

NAME	p-val	FDR qval
KEGG_ANTIGEN_PROCESSING_AND_PRESENTATION	0	0.001771
KEGG_NATURAL_KILLER_CELL_MEDIATED_CYTOTOXICITY	0.001927	0.024245
KEGG_AUTOIMMUNE_THYROID_DISEASE	0.007813	0.050245
KEGG_SYSTEMIC_LUPUS_ERYTHEMATOSUS	0.009881	0.05225
KEGG_GRAFT_VERSUS_HOST_DISEASE	0.00611	0.04818
KEGG_LEISHMANIA_INFECTION	0.012	0.052018
KEGG_ASTHMA	0.009843	0.051692
KEGG_INTESTINAL_IMMUNE_NETWORK_FOR_IGA_PRODUCTION	0.017751	0.062052
KEGG_AMINO_SUGAR_AND_NUCLEOTIDE_SUGAR_METABOLISM	0.002041	0.05631
KEGG_TOLL_LIKE_RECEPTOR_SIGNALING_PATHWAY	0.011976	0.061629
KEGG_HEMATOPOIETIC_CELL_LINEAGE	0.015296	0.064904
KEGG_TYPE_I_DIABETES_MELLITUS	0.017787	0.060479
KEGG_VIRAL_MYOCARDITIS	0.014028	0.059515
KEGG_CYTOSOLIC_DNA_SENSING_PATHWAY	0.003968	0.068157
KEGG_ALLOGRAFT_REJECTION	0.010081	0.064711
KEGG_FRUCTOSE_AND_MANNOSE_METABOLISM	0.026157	0.0754
KEGG_P53_SIGNALING_PATHWAY	0.012024	0.078547
KEGG_PANTOTHENATE_AND_COA_BIOSYNTHESIS	0.006048	0.078132
KEGG_CYTOKINE_CYTOKINE_RECEPTOR_INTERACTION	0.043726	0.111304
KEGG_NOD_LIKE_RECEPTOR_SIGNALING_PATHWAY	0.025641	0.111987
KEGG_APOPTOSIS	0.025948	0.115454
KEGG_PANCREATIC_CANCER	0.025263	0.118664
KEGG_RIG_I_LIKE_RECEPTOR_SIGNALING_PATHWAY	0.035294	0.145911
KEGG_PROTEIN_EXPORT	0.068762	0.140844
KEGG_PRION_DISEASES	0.044776	0.147001
KEGG_ONE_CARBON_POOL_BY_FOLATE	0.05336	0.144019
KEGG_CELL_CYCLE	0.08125	0.166653
KEGG_SPHINGOLIPID_METABOLISM	0.045908	0.171279
KEGG_CHEMOKINE_SIGNALING_PATHWAY	0.075547	0.169606
KEGG_OOCYTE_MEIOSIS	0.068687	0.164303
KEGG_DNA_REPLICATION	0.0998	0.169833
KEGG_GLYOXYLATE_AND_DICARBOXYLATE_METABOLISM	0.070565	0.175985
KEGG_JAK_STAT_SIGNALING_PATHWAY	0.067179	0.19455
KEGG_GALACTOSE_METABOLISM	0.064777	0.19121
KEGG_T_CELL_RECEPTOR_SIGNALING_PATHWAY	0.102161	0.186753
KEGG_GLYCOSAMINOGLYCAN_BIOSYNTHESIS_KERATAN_SULFATE	0.10101	0.187394
KEGG_HOMOLOGOUS_RECOMBINATION	0.112016	0.19811
KEGG_FC_EPSILON_RI_SIGNALING_PATHWAY	0.098837	0.235505
KEGG_AMYOTROPHIC_LATERAL_SCLEROSIS_ALS	0.075547	0.250002
KEGG_ETHER_LIPID_METABOLISM	0.085066	0.245292
KEGG_VEGF_SIGNALING_PATHWAY	0.102823	0.271902

KEGG_CELL_ADHESION_MOLECULES_CAMS	0.171079	0.26877
KEGG_NUCLEOTIDE_EXCISION_REPAIR	0.191617	0.266856
KEGG_PROTEASOME	0.227545	0.277802
KEGG_GLYCOSPHINGOLIPID_BIOSYNTHESIS_LACTO_AND_NEOLACTO_SERIES	0.149897	0.278888
KEGG_GLYCOLYSIS_GLUCONEOGENESIS	0.147475	0.27302
KEGG_PYRIMIDINE_METABOLISM	0.162325	0.268264
KEGG_PRIMARY_IMMUNODEFICIENCY	0.201581	0.278731
KEGG_BLADDER_CANCER	0.14557	0.274716
KEGG_CYSTEINE_AND_METHIONINE_METABOLISM	0.137331	0.2857
KEGG_MAPK_SIGNALING_PATHWAY	0.150101	0.292467
KEGG_PURINE_METABOLISM	0.151052	0.291814
KEGG_PROGESTERONE_MEDIATED_OOCYTE_MATURATION	0.167689	0.288275
KEGG_GLIOMA	0.183128	0.313611
KEGG_COLORECTAL_CANCER	0.174442	0.312966
KEGG_GLYCOSAMINOGLYCAN_BIOSYNTHESIS_CHONDROITIN_SULFATE	0.243299	0.337829
KEGG_PROSTATE_CANCER	0.196	0.334579
KEGG_BASE_EXCISION_REPAIR	0.246063	0.335232
KEGG_COMPLEMENT_AND_COAGULATION_CASCADES	0.248062	0.338573
KEGG_INOSITOL_PHOSPHATE_METABOLISM	0.246436	0.333194
KEGG_FC_GAMMA_R_MEDIATED_PHAGOCYTOSIS	0.264463	0.335231
KEGG_NITROGEN_METABOLISM	0.202479	0.336377
KEGG_ALANINE_ASPARTATE_AND_GLUTAMATE_METABOLISM	0.213693	0.334495
KEGG_GLYCINE_SERINE_AND_THREONINE_METABOLISM	0.246493	0.336135
KEGG_ARGININE_AND_PROLINE_METABOLISM	0.221328	0.334264
KEGG_SNARE_INTERACTIONS_IN_VESICULAR_TRANSPORT	0.246377	0.332902
KEGG_B_CELL_RECEPTOR_SIGNALING_PATHWAY	0.277014	0.3287
KEGG_RENAL_CELL_CARCINOMA	0.261663	0.330464
KEGG_MISMATCH_REPAIR	0.320866	0.339452
KEGG_CHRONIC_MYELOID_LEUKEMIA	0.261224	0.342027
KEGG_PATHWAYS_IN_CANCER	0.269618	0.348573
KEGG_SMALL_CELL_LUNG_CANCER	0.311508	0.382805
KEGG_ALZHEIMERS_DISEASE	0.316206	0.380826
KEGG_NON_SMALL_CELL_LUNG_CANCER	0.316116	0.391053
KEGG_ALDOSTERONE_REGULATED_SODIUM_REABSORPTION	0.317365	0.403454
KEGG_LEUKOCYTE_TRANSENDOTHELIAL_MIGRATION	0.356275	0.408845
KEGG_LONG_TERM_POTENTIATION	0.348162	0.412247
KEGG_RNA_DEGRADATION	0.371486	0.411294
KEGG_REGULATION_OF_ACTIN_CYTOSKELETON	0.360248	0.413197
KEGG_HUNTINGTONS_DISEASE	0.391667	0.412768
KEGG_RNA_POLYMERASE	0.375746	0.416831
KEGG_TYPE_II_DIABETES_MELLITUS	0.371951	0.416821
KEGG_LONG_TERM_DEPRESSION	0.374257	0.420801
KEGG_PORPHYRIN_AND_CHLOROPHYLL_METABOLISM	0.394892	0.416656
KEGG_BASAL_TRANSCRIPTION_FACTORS	0.388655	0.420075

KEGG_GLYCOSAMINOGLYCAN_DEGRADATION	0.396414	0.425598
KEGG_BETA_ALANINE_METABOLISM	0.41483	0.424407
KEGG_PATHOGENIC_ESCHERICHIA_COLI_INFECTION	0.421505	0.444809
KEGG_PHENYLALANINE_METABOLISM	0.471774	0.478344
KEGG_PARKINSONS_DISEASE	0.484	0.483796
KEGG_GLYCOSAMINOGLYCAN_BIOSYNTHESIS_HEPARAN_SULFATE	0.482255	0.479549
KEGG_LINOLEIC_ACID_METABOLISM	0.557312	0.572343
KEGG_REGULATION_OF_AUTOPHAGY	0.626953	0.587292
KEGG_O_GLYCAN_BIOSYNTHESIS	0.662028	0.662246
KEGG_SPLICEOSOME	0.612403	0.681184
KEGG_FOCAL_ADHESION	0.678647	0.755411
KEGG_TERPENOID_BACKBONE_BIOSYNTHESIS	0.757576	0.795974
KEGG_OXIDATIVE_PHOSPHORYLATION	0.767396	0.848829
KEGG_ECM_RECEPTOR_INTERACTION	0.760649	0.879225

Supplementary Table 1: Overlapped genes were identified between DEGs in the different TMB level groups and the IRGs obtained from ImmPort database

Type	Gene symbol
overlapped genes	AZGP1 CD8A CD74 CTSB CTSE FCER1G FCGRT HLA-DMA HLA-DMB HLA-DOA HLA-DPA1 HLA-DPB1 HLA-DQA1 HLA-DQA2 HLA-DQB1 HLA-DRA HLA-DRB1 HLA-DRB5 HSPA2 HSPA6 ICAM1 IFNG KIR2DL4 KLRC2 CIITA MICB PSME1 PSME2 TAP1 TAP2 IFI30 CD209 ULBP3 ULBP2 RAET1E PI3 REG3G CXCL14 CXCL16 CXCL10 CXCL9 CXCL5 CXCL11 CXCL1 CXCL13 PF4 CXCL3 CCL13 DEFB1 CCL8 COLEC10 S100A8 S100A12 MMP12 TMSB15A SERPIND1 S100P S100A2 LCN12 S100A14 S100A16 IL6 TGFB1 PF4V1 APOBEC3G FABP6 NOD2 RBP1 TLR2 PLAU IL1B PAEP MUC5AC PLTP MX1 DDX58 LBP R3HDML OASL CRABP1 OBP2B RBP2 LCN15 C8G NOX1 APOD ORM2 PML CYBB ISG20 DUOX2 TLR3 IFIH1 LRP1 IDO1 STAT1 SOCS3 SEMG1 SOCS1 IRF1 IL15 APOBEC3F RARRES3 CHIT1 ISG15 DHX58 MUC4 ELN LYZ CCL5 CST4 JUN TLR8 GNLY BST2 PLA2G2A NOS2 TLR1 FGA MSR1 ROBO3 SLC11A1 DMBT1 TNFRSF10A CCL4 APOBEC3H TMPRSS6 MARCO TNFSF11 CLDN4 IL17A APOBEC3A IL7R SYTL1 PTGS2 CD14 PROC TRIM22 RSAD2 PDCD1 AQP9 FASLG GBP2 CCL18 CCL26 CCL4L2 CCL7 CCL3 CCR5 CCL3L1 CCR1 CCL24 XCL2 CXCR4 CXCR6 IL10 PPARG FGR JAK2 CD86 HCK OLR1 RNASE2 LYN VAV3 RAC2 RAC3 CHP2 NFATC1 NFATC2 CARD11 PIK3R5 PIK3CD CD72 LILRB3 FCGR2B CX3CL1 EDN1 PROK2 SAA1 SAA2 SEMA3C SEMA4B SEMA5A TNC TYMP C5AR1 CMKLR1 EDNRB FPR1 FPR2 GPR17 PLXNB1 PLXNC1 PTAFR ROBO2 ADM AGT AMELX AMH AREG BMP3 BMP5 BMP7 CALCA CCK CD70 CHGA CHGB CMTM2 CSF1 CSF2 CSPG5 DKK1 EREG FAM3B FAM3D FGF18 FGF20 FGF3 GCG GDF10 GDNF GREM2 GRP GUCA2A IGF2 IL17C IL1RN IL24 IL34 INHBB INSL5 KL LEFTY1 LEFTY2 NODAL NRG1 NRTN NTS NUDT6 OSM PDGFD PDGFRL REG1A RETN RETNLB RLN2 SCT SECTM1 SPP1 STC1 TAC1 TDGF1 TG TNFSF13B TNFSF14 TNFSF9 UCN2 ADRB1 AVPR1A C3AR1 CSF1R CSF2RB CSF3R EPOR FGFR2 FGFR3 FGFR4 GHR GIPR HNF4A IL10RA IL12RB1 IL18R1 IL18RAP IL1R2 IL21R IL22RA1 IL23R IL2RA IL2RB LGR5 MTNR1A NPR3 NR1I2 NR4A2 NR6A1 NRP1 PRLR PTGER1 PTGER2 RARA S1PR2 SSTR5 TGFBR2 THRB TNFRSF10D TNFRSF11A TNFRSF18 TNFRSF19 TNFRSF9 VIPR1 ITGAL ITGB2 TYROBP LCK FCGR3A FCGR3B CD247 LCP2 PLCG1 FYN HCST CD48 CD244 PRKCG SH2D1A FAS PRF1 CD3D CD3E CD3G PTPRC ITK NCK2 ICOS CTLA4 RASGRP1

Supplementary Figure 1: Kaplan-Meier survival curves showed no statistically significant differences between the high- and low-TMB groups.

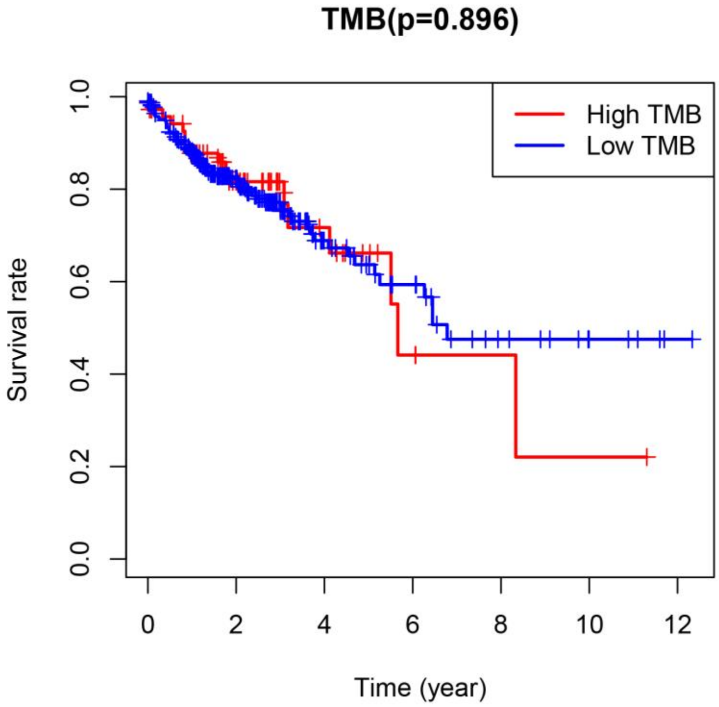

Supplement: Supplementary file 1 — Supplementary figure and tables. [file ijmsv18p0685s1.pdf]
